# What are the virtual reality solutions for dual-task intervention to promote health in aging? A scoping review

**DOI:** 10.3389/fnhum.2025.1753364

**Published:** 2026-01-28

**Authors:** Francesca Bruni, Francesco Bigotto, Valentina Mancuso, Silvia Cavedoni, Jonathan Panigada, Marco Stramba-Badiale, Silvia Serino, Elisa Pedroli

**Affiliations:** 1 Older People Rehabilitation and Cerebrovascular Medicine Research Laboratory, IRCCS Istituto Auxologico Italiano, Milan, Italy; 2 Department of Theoretical and Applied Sciences, eCampus University, Novedrate, Italy; 3 Department of Medicine, Neurology and Rehabilitation, IRCCS Istituto Auxologico Italiano, Milan, Italy; 4 Department of Psychology, University of Milano-Bicocca, Milan, Italy; 5 Bicocca Center for Applied Psychology, University of Milano-Bicocca, Milan, Italy

**Keywords:** aging, assessment, dual-task, rehabilitation, technology, virtual reality

## Abstract

**Introduction:**

Cognitive-motor dual-task (CMDT) performance is crucial for everyday activities, particularly in older adults who commonly experience deficiencies in cognitive and motor domains. These impairments compromise daily functioning, causing a decrease in quality of life. However, traditional assessment and training methods face significant limitations. Conventional protocols often exhibit limited ecological validity, as they fail to replicate real-world performance demands adequately. Moreover, they are characterized by substantial methodological heterogeneity and offer restricted capacity for real-time performance monitoring and the delivery of personalized feedback. Virtual Reality (VR) offers a promising approach by creating controlled, real-life environments that enable precise assessment and personalized training. However, VR-based CMDT remains largely unexplored. This review evaluates current VR solutions for CMDT, examining assessment, and intervention designs.

**Methods:**

A scoping review was conducted on April 17, 2023 (and updated on January 21, 2025), following the PRISMA for Scoping Review guidelines, on three databases: Web of Science, Embase, and PubMed. We included original articles, based on the following criteria: English-language; original research articles reporting empirical data from experimental, quasi-experimental, or observational studies; articles employing CMDT assessment or training; use of VR-based applications during a CMDT; older population participants (≥65 years). Reviews, meta-analyses, editorials, conceptual articles, case studies, and short papers were excluded.

**Results:**

Of 2,649 papers, 21 studies met the inclusion criteria: 17 assessment and four training studies. Research focused on healthy older adults and individuals with early cognitive decline, sensory deficits, and those at fall risk. Assessment studies mainly employed immersive and semi-immersive systems simulating realistic scenarios in virtual environments, primarily using “walking while” paradigms with cognitive tasks. Training studies frequently utilized non-immersive or semi-immersive solutions with less naturalistic environments, demonstrating improvements in gait parameters, dual-task performance, motor functions, and cognitive abilities. Substantial methodological heterogeneity was observed in intervention duration, frequency, and DT cost calculations, with limited attention to user experience evaluation.

**Discussion:**

VR-based CMDT applications show promise for assessment and intervention in aging. However, significant gaps exist regarding the lack of standardized methodological approaches, ecological validity, and user-centered design considerations. Future research should address these limitations to enhance the accessibility and effectiveness of VR-based CMDT interventions.

## 1 Introduction

The intersection of cognitive and motor functions represents a critical area for understanding age-related functional decline, particularly since daily activities often require simultaneous performance of multiple tasks that compete for shared cognitive resources. Research has consistently demonstrated that dual-task (DT) performance predicts real-world functional outcomes (Schubert et al., 2017; Strobach et al., 2012), yet traditional assessment and intervention approaches face significant limitations in addressing the complex cognitive-motor interactions involved. Specifically, conventional protocols may lack ecological validity, failing to simulate real-world demands, demonstrate substantial methodological heterogeneity in task selection and outcome measurement (Leone et al., 2015), and provide limited capacity for real-time performance monitoring and personalized feedback. Virtual Reality (VR) emerges as a promising technological advancement offering unique capabilities for creating ecologically valid, controlled environments that enable precise assessment and personalized training of cognitive-motor dual-task (CMDT) performance.

This scoping review aims to provide a comprehensive perspective on the intersection of VR technology, CMDT task used in assessment and rehabilitation fields, and gerontology, presenting the state-of-the-art in this panorama. Initially, we elucidate the critical importance of focusing programs on CMDT. We then discuss the rationale for implementing VR technology in this domain and examine its benefits and drawbacks. The paper provides a comprehensive overview of the assessment and training instruments used, detailing the types of interventions and VR solutions to guide future research and clinical applications. We analyse literature, providing insights into the impacts and challenges of interventions using new technology for older individuals. The presentation of the results is followed by a future-oriented comment offering an opportunity to identify research gaps, suggesting methodological improvements, and proposing new directions for VR-based CMDT interventions in this rapidly evolving field.

### 1.1 Cognitive-motor dual-task: assessment and rehabilitation approaches

As described by McIsaac et al. (2015), DT refers to the concurrent performance of two tasks that can be executed independently and measured separately, having distinct goals. DT is typically categorized into three types, based on the nature of the tasks being performed: motor-motor, cognitive-cognitive, and cognitive-motor dual-task (Piqueres-Juan et al., 2021). Motor-motor and cognitive-cognitive DT involves two tasks performed concurrently, whether motor (e.g., as walking while playing an instrument) or cognitive (e.g., counting backward while categorizing figures), respectively. CMDT involves one motor task and one cognitive task performed concurrently, such as balancing while engaging in working memory tasks. While all three configurations appear frequently in daily contexts, recent research has predominantly focused on CMDT, which represents a critical domain for understanding how cognitive functions affect motor performance and vice versa (Li et al., 2018; Postigo-Alonso et al., 2019). CMDT requires sophisticated integration of multiple complex skills, including strategic planning, task implementation, performance monitoring, working memory allocation, and attentional modulation. However, the cognitive capacity to allocate resources across multiple tasks is inherently limited, resulting in performance decrements that manifest as reduced performance quality and increased task completion time, regardless of individual competencies (MacPherson, 2018). These issues become even more notable with aging, where older adults require significantly more attentional resources to complete tasks due to both cognitive and motor decline (García-López et al., 2023; Tuena et al., 2023; Versi et al., 2022), as supported by neuroimaging evidence revealing age-related structural and functional changes, particularly in frontal regions, that impact higher-level cognitive functions (Andrews-Hanna et al., 2007; Grady, 2012; Raz et al., 2005). These cognitive decrements correlate with impaired mobility parameters, including gait velocity reduction and fall risk (Yogev-Seligmann et al., 2008), potentially compromising functional independence (Wajda et al., 2017). In pathological conditions, cognitive-motor interference exceeds isolated domains (Li et al., 2018; Wollesen and Voelcker-Rehage, 2014). For instance, disorders related to pathological aging (e.g., Parkinson's disease and Mild Cognitive Impairment) demonstrate particularly pronounced DT costs, with patients exhibiting disproportionate performance deterioration compared to healthy age-matched controls due to compromised neural networks and impaired automaticity of motor and cognitive processes (Al-Yahya et al., 2011; Yogev-Seligmann et al., 2008).

Given these clinical implications, an accurate assessment of CMDT performance becomes crucial. Typically, assessment design integrates both motor detections (e.g., spatiotemporal gait parameters during standardized walking tasks) and cognitive challenges under single and DT conditions. Established protocols include the cognitive Timed Up and Go test (cognitive-TUG), where participants perform the standard Timed Up and Go test (Podsiadlo and Richardson, 1991) while engaging in cognitive tasks such as counting backward, generating animal names, or spelling given words backward (Kasiukiewicz et al., 2021; Shumway-Cook et al., 2000). Another widely used protocol is the Walking While Talking test, involving walking at a preferred pace during concurrent cognitive tasks of varying complexity (Yogev-Seligmann et al., 2008). Performance variations in both domains (i.e., DT cost)—manifested as the difference in performance between single-task and DT conditions—provide quantitative indices of cognitive-motor interference. The assessment of CMDT offers valuable insights for the detection of emerging cognitive-motor problems and facilitates timely individualized intervention (Mancioppi et al., 2021). This assessment foundation translates into therapeutic applications, as CMDT performance demonstrates significant trainability through targeted interventions.

The CMDT training, integrating simultaneous performance of cognitive tasks (e.g., verbal fluency, memory exercises) with motor activities (walking, balance exercises) (Fritz et al., 2015; Piqueres-Juan et al., 2021; Tuena et al., 2023), has proven as a valuable approach for enhancing cognitive and motor functions across healthy aging and pathological populations (Wiśniowska et al., 2023; Wongcharoen et al., 2017; García-López et al., 2023; Li et al., 2020; Tait et al., 2017; Versi et al., 2022). Despite literature providing several methods to deliver CMDT (i.e., sequential or simultaneous task performance), simultaneous training, performing motor and cognitive tasks at the same time, seems to be the most effective in improving balance, gait, and reducing fall risks in older adults with chronic conditions (Spanò et al., 2022). The effectiveness of these interventions is attributed to the activation and strengthening of compensatory neural mechanisms, where alternative neural circuits develop to support cognitive-motor function despite age-related changes (Miura et al., 2024; Park and Reuter-Lorenz, 2009). However, current CMDT training approaches face several significant limitations that restrict their clinical translation and effectiveness, such as the optimal dose-response. This parameter remains undefined, with training protocol varying widely from 2–3 sessions per week lasting 30–60 min over a period of 4–12 weeks (Forte et al., 2023; Petrigna et al., 2025). Additionally, these limitations extend to CMDT evaluation methodologies, where there is no consensus on an optimal evaluation method, with protocols varying considerably across studies in terms of task selection and outcome measures, limiting meaningful comparison of results and clinical applicability (Leone et al., 2015). Most CMDT protocols also remain anchored to traditional methods with minimal incorporation of advanced technologies that could enhance engagement, precision, and objective monitoring. Moreover, actual CMDT options may fail to capture real abilities in an ecological context because the stimuli often do not reflect real-life activities.

### 1.2 Why is virtual reality an added value?

During the history of neuropsychology, attention has shifted from traditional paper-and-pencil methods to computer-based programs, offering multimodal and customizable instruments (Hu et al., 2021). Contemporary approaches fundamentally maintain the same theoretical foundations as conventional training, with the primary innovation being the integration of gamification elements and interactive technologies that enhance engagement and motivation (Bieryla and Dold, 2013; Eggenberger et al., 2016; Hu et al., 2021; Nouchi et al., 2012; Rendon et al., 2012). Among these technological advances, VR emerges as a particularly transformative tool for CMDT, offering a unique advantage in creating realistic tasks while enabling multisensory bodily interactions (Riva et al., 2018). The effectiveness of VR depends on several key features: the sense of presence within the environment (the sensation of “being there”), agency (active participation), and immersion (sensory fidelity level) (Cipresso et al., 2018; Riva et al., 2020; Slater, 2009). Based on these features, the literature conceptualized VR along a continuum of levels (Borghesi et al., 2022): fully immersive systems (e.g., Cave environments with body tracking, head-mounted displays) offering complete isolation from the real world and direct virtual interaction; semi-immersive systems (e.g., large-screen projections with tracking capabilities) providing moderate immersion; and non-immersive systems (e.g., traditional screens with motion sensors, console games with motion controllers) delivering a degree of immersion and interaction beyond traditional screens yet fall short of fully immersive experiences.

Whatever its form, VR offers significant advantages to address limitations in traditional CMDT: reproducible, ecologically valid evaluation environments, such as virtual supermarkets, home environments (Da Costa et al., 2022; Raspelli et al., 2011), or virtual towns (Da Costa et al., 2022) that can be deployed across different clinical settings and aging populations. This reproducibility provides the infrastructure necessary for standardization, which requires researchers to converge on shared protocols beyond technical implementation. VR systems enable automated, objective performance tracking that captures both cognitive and motor parameters simultaneously, reducing measurement variability and facilitating precise DT cost calculations (Tuena et al., 2023; Wei et al., 2025). Moreover, VR enhances cost and temporal efficiencies, safety protocols, and accessibility (Wei et al., 2025) while creating engaging scenarios that promote transfer to daily activities within controlled laboratory conditions (Yun et al., 2023). Finally, when training is delivered via VR-based interventions, it demonstrates significant improvements in outcomes compared to traditional training methods (Wei et al., 2025), indicating not only methodological advantages but also enhanced clinical effectiveness. Nevertheless, VR implementation faces important practical challenges, particularly for older populations. Cybersickness (i.e., nausea, dizziness, disorientation, and general discomfort, depending on system characteristics) may potentially pose a risk to older adults due to age-related vestibular and visual changes (Rebenitsch and Owen, 2016). Hardware cost and technical complexity can limit accessibility for resource-constrained settings and home-based interventions (Wei et al., 2025). Technological literacy barriers may require extended familiarization and ongoing support, while safety concerns include fall risk during immersive experiences for individuals with balance impairments. Usability issues may further compromise feasibility (Realdon et al., 2019; Tuena et al., 2023). These considerations necessitate careful system selection and user-centered design that prioritize safety, comfort, and accessibility alongside technical capabilities.

Recent systematic reviews examined CMDT interventions assisted with technologies, focusing either on aging population with chronic diseases (Tuena et al., 2023) or on healthy older adults (Wei et al., 2025). However, these reviews have focused predominantly on training applications, with CMDT assessment methodologies receiving comparatively less systematic attention despite their crucial role in designing tailored interventions (Choi et al., 2025; Tuena et al., 2023; Wei et al., 2025). Furthermore, literature also lacks a systematic comparison across different levels of VR immersion (non-immersive, semi-immersive, and fully immersive) within the CMDT context. Moreover, emerging technological innovation (such as 360° media), which has shown promise in other cognitive assessment domains (Mancuso et al., 2024), may offer an additional solution for CMDT applications. Although their current use in this context remains unclear. The present scoping review addresses these gaps by mapping both assessment and intervention applications across the full aging spectrum (≥65), with particular attention to: (i) characterizing the spectrum of VR immersion levels currently employed (non-immersive, semi-immersive, fully immersive) and their relationship with ecological validity, (ii) providing a comprehensive view integrating both assessment and training applications of VR-based CMDT, and (iii) identifying which technological approaches are currently utilized and which promising innovations remain unexplored, as well as methodological inconsistencies that limit clinical translation.

## 2 Methods

Given our objective of identifying, mapping, reporting, and discussing the key characteristics and concepts of VR-based CMDT interventions, we determined that a scoping review methodology was the most appropriate approach. This methodology allows for a comprehensive exploration of the literature, facilitating a broad overview of the field and enabling us to synthesize diverse intervention characteristics effectively, identifying and analyzing knowledge gaps (Munn et al., 2018).

### 2.1 Literature search

This scoping review was conducted on April 17, 2023 (and updated on January 21, 2025) and reported according to the Preferred Reporting Items for Systematic Reviews and Meta-Analyses extension for Scoping Reviews (PRISMA-ScR) guidelines (Tricco et al., 2018). No protocol was registered in advance, consistent with the exploratory nature of scoping reviews (Munn et al., 2018; Tricco et al., 2018). We performed a computer-based search in three databases: PubMed, Web of Science, and Embase. These databases were selected to provide multidisciplinary coverage relevant to VR-based interventions in aging populations. Web of Science indexes content across psychology, engineering, and rehabilitation sciences; Embase provides comprehensive coverage of biomedical and allied health journals; and PubMed ensures thorough capture of medical and clinical research. The literature review methodology incorporated two distinct search strings, each with a specific focus. The first tackled assessment methodologies, while the second addressed rehabilitation strategies:

- (aging OR elder^*^ OR old OR patient^*^ OR olde^*^) AND (“dual task” OR dual-task OR cognitive-motor) AND (360° video^*^ OR 360° image^*^ OR equirectangular image^*^ OR equirectangular video^*^ OR 360-degree video^*^ OR 360-degree image^*^ OR spheri^*^ video^*^ OR 360° technology OR 360-degree technology OR 360 technology OR 360 degree technology OR immersive video^*^ OR immersive image^*^ OR 360 degree medi^*^ OR virtual reality OR VR OR virtual-based OR virtual OR immersive OR simul^*^) AND (assessment OR assess^*^ OR evaluat^*^ OR evaluation OR diagnos^*^).
- (aging OR elder^*^ OR old OR older^*^ OR patient) AND (“dual task” OR dual-task OR cognitive-motor) AND (“360° video^*^” OR “360° image^*^” OR “equirectangular image^*^” OR “equirectangular video^*^” OR “360-degree video^*^” OR “360-degree image^*^” OR “spheri^*^ video^*^” OR “360° technology” OR “360-degree technology” OR “360 technology” OR “360 degree technology” OR “immersive video^*^” OR “immersive image^*^” OR “360 degree medi^*^” OR “virtual reality” OR “VR” OR “virtual-based” OR virtual OR immersive OR simul^*^) AND (rehabilitation OR treatment OR rehab^*^ OR management OR therapy OR training OR intervention).

The term “patient” was strategically included alongside aging-related keywords to encompass physiological aging and older clinical populations, aligning with established conventions in scientific literature. Similarly, we included terminology related to 360° media in our search strategy, although this represents an emerging frontier in this field. This inclusive approach ensured comprehensive coverage of literature, preventing the omission of relevant research.

The search strings were applied to title and abstract fields in PubMed and Embase, and to the Topic field (which includes title, abstract, author keywords, and Keywords Plus) in Web of Science. Search strategies were adapted to each database's specific syntax while maintaining semantic equivalence: in PubMed, we used field tags [Title/Abstract]; in Embase, we applied.tw. (text word) for title, abstract, and keyword fields; in Web of Science, we searched the Topic field, which includes title, abstract, author keywords, and Keywords.

### 2.2 Selection criteria

The following hierarchy of eligibility criteria was adopted for two-stage screening: title/abstract and full-text:

1. Articles in English;

2. Original research articles reporting empirical data from experimental, quasi-experimental, or observational studies. Reviews, meta-analyses, systematic reviews, editorials, commentaries, conceptual articles, case studies, and short papers were excluded;

3. Articles that defined their intervention/assessment method as a CMDT (with methods either thoroughly described or properly referenced);

4. Articles with use of VR during cognitive-motor dual task;

5. Articles with average age of the population 65 years or older.

Studies were excluded if they: (i) described exclusively technical aspects or VR prototypes without reporting any data from human participants performing CMDT; (ii) employed VR in a purely descriptive or proof-of-concept manner without any quantitative or qualitative measurements of CMDT performance, cognitive outcomes, motor outcomes, or user experience; (iii) focused solely on system development or technical validation without assessing behavioral or functional measures during DT conditions.

### 2.3 Data extraction and synthesis

The selected articles were assigned to four independent researchers (JP, FB, VM, and SC), who worked in pairs to select the data using a standardized form. We included an additional researcher (FBr) who collected the data for every study and then verified its accuracy and completeness. Conflicts were resolved by consensus within each pair or by involvement of the fifth author when necessary. Each pair screened all records by title and abstract. Articles meeting initial criteria advanced to full-text assessment, where the same paired-reviewer process was applied. Reasons for exclusion at both stages of screening were documented according to the five selection criteria outlined above.

Consistent with scoping review methodology (Munn et al., 2018), we did not conduct a formal quality assessment or risk of bias appraisal, as we aimed to map the breadth and characteristics of VR-based CMDT approaches rather than to synthesize intervention effectiveness.

## 3 Results

**Figure 1:**
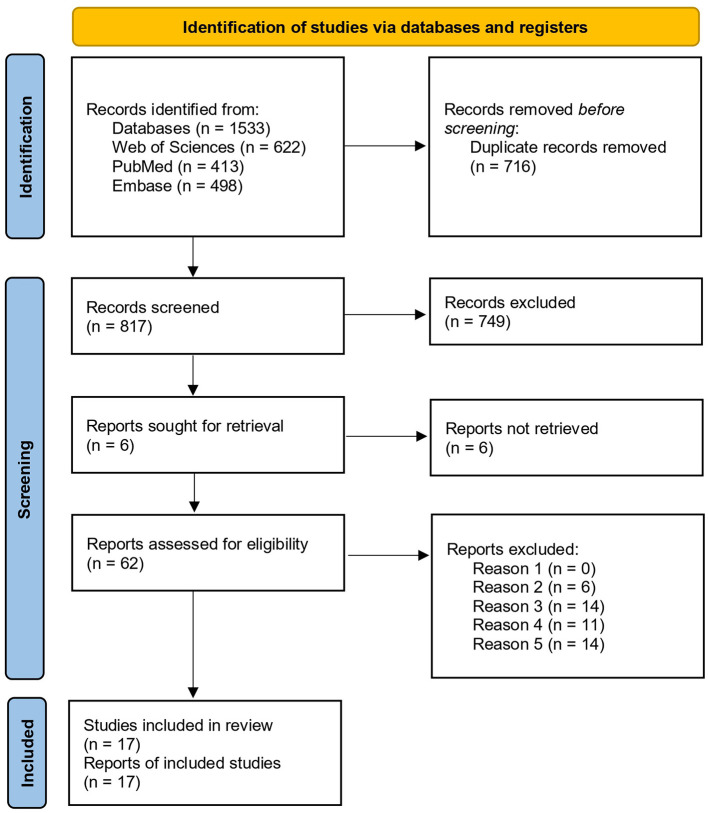
PRISMA flowchart of the included studies using terms related to the assessment.

**Figure 2:**
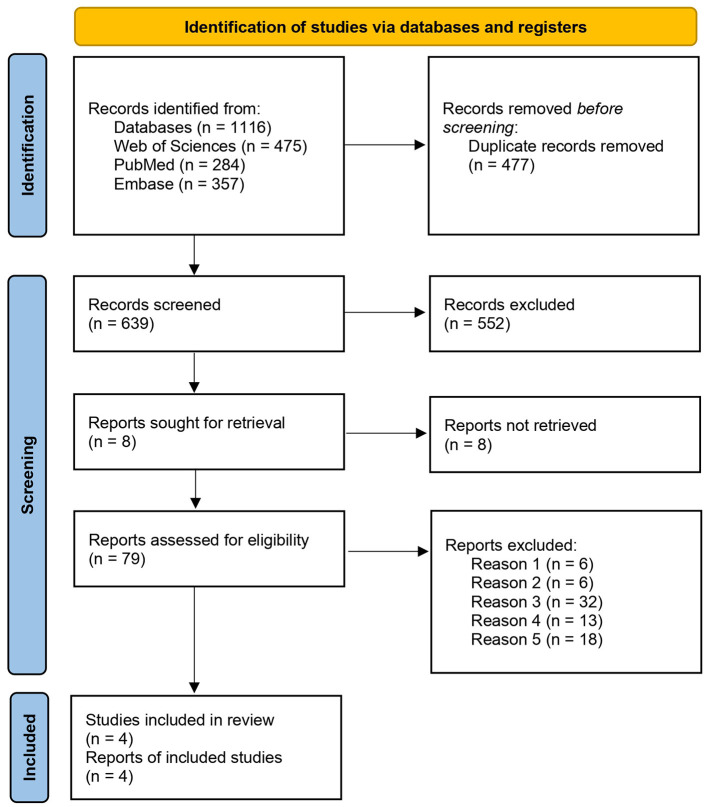
PRISMA flowchart of the included studies using terms related to the rehabilitation field.

The initial search yielded 1,533 citations screened with VR-based CMDT assessment focus and 1,116 with rehabilitation, including duplicate studies. After the screening processes, papers were reduced to 17 and 4 articles, respectively. A flow diagram showing the procedure is detailed in Figure 1 for literature focused on assessment tools and in Figure 2 for those related to rehabilitation (Page et al., 2021).

We extracted and analyzed key variables from the selected studies, and the results are summarized in Tables 1, 2. The following paragraphs address three specific research questions that emerged from gaps identified in the literature: (i) what characterizes VR-based CMDT approaches in older adults? (ii) How do technological choices relate to ecological validity? (iii) Are there methodological limitations?

**Table 1:** Summary of the included studies about CMDT assessment using VR systems.

**Study**	**Participants**	**Adopted technology**	**DT description**	**Principal outcomes**	**Virtual environment**
Carr et al. (2020)	14 older adults	StreetLab: a VR laboratory composed of a curved projection screen combined with sound loudspeakers, a subwoofer, and a strain gage force plate (immersive)	Listening task (three conditions of the Dichotic Digit Test), while either standing on a firm surface or on a Balance-Pad placed on top of the force plate (compliant surface), or sitting on a chair, with a concurrent visual stimulation in VR	(i) Listening performance (spoken word recognition accuracy); (ii) motor functions (postural performance via center of pressure path length); (iii) DT cost (percentage change in performance from single task to DT conditions)	High-resolution static simulation of a street intersection in downtown Toronto
Ehlers et al. (2017)	195 older adults	CAVE Automatic Virtual Environment with treadmill (immersive)	Walking on a self-propelled treadmill and safely crossing a virtual street while conversing with a researcher using a hands-free cell phone	(i) Street crossing performance (DT and single-task)	Virtual street. The street included two lanes of cars traveling free of extraneous obstacles, and did not include any safe zones where participants could wait for cars to pass
Tung Lau et al. (2016)	Eight older adults with bilateral hearing loss with hearing aids, and eight controls with normal hearing thresholds	StreetLab: a VR reality laboratory composed of a curved projection screen combined with sound loudspeakers, a subwoofer, and a strain gage force plate (immersive)	Recognizing words spoken by a target speaker when there was a competing talker (80-word recognition trials), while crossing a virtual street on a treadmill	(i) Listening performance (word recognition accuracy); (ii) kinematic parameters (head and trunk angles, step width and length, stride time, cadence); (iii) street crossing performance (iv) DT cost (subtraction of the DT condition results from the single-task performance)	Downtown street intersection with six lanes of two-directional traffic with a median strip
Cook et al. (2013)	Forty-six healthy and 15 memory-impaired adults	Drive software (STISIM) running on a computer desktop with Logitech MOMO Force Feedback Steering Wheel as controller (semi-immersive)	Hearing, briefly mentally rehearsing, then verbally recalling (paragraph recall memory task) a short story while driving in a simulated lane navigation driving task	(i) Cognitive functions (memory: accuracy and completeness of story recalling); (ii) Driving performance (lane navigation);(iii) DT cost (ANOVA comparing single and DT performances)	Straight or curved roadway
Francis et al. (2015)	Eleven healthy older adults	A two-belt treadmill with a semi-circular rear-projection screen with a passive motion capture system (semi-immersive)	Walking in a virtual hallway while counting backwards by seven, starting from a three-digit number	(i) Cognitive functions (correct counting); (ii) motor functions (step width and length); (iii) DT cost (ANOVA comparing single and DT performances)	A virtual hallway moving at the same speed as the treadmill, with an added sinusoid moving on the side to distract
Stojan et al. (2023)	Fifty-six healthy older adults	GRAIL system: a 3D instrumented split-belt treadmill with two embedded force plates, integrated with a semi-cylindrical 240° projection screen (semi-immersive)	Serial Threes Task while walking, or Color Word Stroop Task while walking	(i) Cognitive functions (reaction time and accuracy—Stroop test; number of correct calculations—Serial 3′s); (ii) motor functions (step time variability of walking; balance integration score); (iii) DT cost (linear mixed models effects comparing single and DT performances)	Industrial-like setting with abstract objects alongside a rectilinear walking path
de Rond et al. (2021)	Seventeen community-dwelling older adults	A 3D-VICON system for capturing motion, with eight reflective markers, six infrared 3D motion capture cameras, and a projector screen (non-immersive)	Wasp game: weight shifting to activate a virtual water jet to hit wasps, while silently performing a serial subtraction task	(i) Motor functions (mediolateral weight shifting for speed and accuracy; number of hits); (ii) cognitive functions (working memory via serial subtraction task); (iii) DT cost (linear mixed models effects comparing single and DT performances)	A wood with a car, the wasp, a street, and a pond
Souza Silva et al. (2020)	Fourteen healthy older adults	HMD equipped with reflective markers and Bluetooth earphones (immersive)	Walking at a comfortable pace toward a blue target and avoiding collision with approaching pedestrians while receiving and reporting text messages delivered at the onset of pedestrian movement or near a pedestrian crossing	(i) Cognitive functions (accuracy of message report; onset time of avoidance strategy); (ii) motor functions (maximum medio-lateral path deviation, walking speed); (iii) obstacle circumvention tasks (minimum distance maintained from the obstacle; number of collisions); (iv) DT cost (percentage change in performance from single task to DT conditions)	A subway station with three virtual pedestrians
Carr et al. (2019)	Sxiteen community-dwelling older adults with subjective cognitive decline, and 14 without any subjective difficulty	StreetLab: a VR laboratory composed of a curved projection screen combined with sound loudspeakers, a subwoofer, and a strain gage force plate (immersive)	Either standing on a firm surface or on a Balance-Pad placed on top of the force plate (compliant surface), or sitting on a chair, with a concurrent visual stimulation in VR while performing three different conditions of a listening task (double-digit numbers repetition)	(i) Listening performance (response accuracy of digit repetition); (ii) motor functions (postural performance via center of pressure path length); (iii) DT costs (ANOVA comparing single and DT performances)	High-resolution static simulation of a street intersection in downtown Toronto
Wu et al. (2014)	Twenty-nine older adults (25 of whom were experienced hearing aid users)	SIREN (Simulator for Research in Ergonomics and Neuroscience): a fixed-based, interactive driving simulator comprised of a running gear removed, electronic sensors, and miniature cameras for recording driver performance, four projectors with image generators, and computers for scenario design, control, and data collection (semi-immersive)	Speech recognition and driving tasks: driving the simulator while listening to/repeating the sentences recorded using different hearing aid settings	(i) Driving performance (following task—distance from the lead vehicle); (ii) speech recognition task (number of keywords repeated); (iii) DT costs (ANOVA comparing single and DT performances)	A car-following scenario on a highway
Gaspar et al. (2013)	Fourteen independent-living older adults with high falls risk, and 14 with low falls risk	General Motors Saturn automobile surrounded by eight screens and a PC for neuropsychological testing (semi-immersive)	Continuous 1-Back task, while driving	(i) Driving performance (following task—distance from the lead vehicle); (ii) cognitive functions (counting accuracy in the 1-back task); (iii) DT costs (ANOVA comparing single and DT performances)	A car-following scenario in a straight two-lane highway
Nagamatsu et al. (2011)	Thirty-three community-dwelling older adults	CAVE Automatic Virtual Environment with treadmill (immersive)	Crossing two lanes without getting hit by oncoming traffic, while listening to one of several pre-made playlists through earphones (Music condition), or while conversing on the phone with an experimenter via a hands-free headset (Phone condition)	(i) Street crossing performance (number of successful crossings; collision vs. timed out unsuccessful trials; first-lane collisions; time to cross the street); (ii) DT costs (ANOVA comparing single and DT performances)	A busy two-way street, with cars approaching from both directions
Lewis et al. (2023)	Ten healthy older adults	HMD, treadmill, and VR tracking devices in the lower back and feet (immersive)	Walking 200 m through a grocery store while searching as quickly and efficiently as possible for five items on the shopping list	(i) VR application performance (trial duration; correct and incorrect items; number and duration of list activations; number and duration of stops; walking speed; words recalled; sale item deliberation); (ii) DT costs (number and duration of stops during VR performance)	A virtual grocery store
Mohanathas et al. (2024)	Twenty-two healthy older adults, and 22 older adults with age-related hearing loss with bilateral hearing aids	StreetLab: a VR laboratory composed of a curved projection screen combined with sound loudspeakers, a subwoofer, and a strain gage force plate (immersive)	Listening and repeating back a series of two or four digits (dichotic digits task) while standing on either a firm or a compliant surface	(i) Motor functions (balance: average center of pressure, mean velocity, and variability measures); (ii) listening performance (digit recognition accuracy); (iii) DT costs (repeated measures ANOVAs comparing single and DT performances)	An immersive, multisensory, VR environment. A 6-lane intersection in Toronto was created using OpenScene Graph
Rosenfeldt et al. (2024)	Fifteen healthy older adults, and 16 with Parkinson's Disease	HMD, treadmill, and VR tracking devices in the lower back and feet (immersive)	Walking 200 m through a grocery store while searching as quickly and efficiently as possible for five items on the shopping list	(i) VR application performance (total trial duration, number of correct items, number of retrieval errors); (ii) biomechanical functions (total movement time, stop time, maximum velocity); (iii) list observation metrics; (iv) cognitive functions (time spent searching for items and deliberating on sale items); (v) DT costs (number of stops, duration of stops, time spent viewing the list, DT gait speed)	A virtual grocery store
Downey et al. (2023)	Twenty healthy older adults, and 20 older adults with hearing impairment who use hearing aids	StreetLab: a VR laboratory composed of a curved projection screen combined with sound loudspeakers, a subwoofer, and a strain gage force plate (immersive)	Standing on a force plate while concurrently performing either an auditory 2-back task (listening working memory performance) or the coordinated response measures task (sentence recognition between two competitive talkers)	(i) Auditory-cognitive functions (accuracy and time reaction); (ii) motor functions (walking— temporal characteristics of gait: step time, stride time; posture: spatial, temporal, and variability measures); (iii) DT costs (percentage change in performance from single task to DT conditions)	The virtual environment used in this study will depict a large urban 6-lane street intersection in Toronto.
Mack et al. (2023)	Fifty healthy older adults	A street crossing simulator: a treadmill and three flat screens that featured a 195-degree horizontal field of view (semi-immersive)	Street crossing while performing two cognitively demanding tasks: using a smartphone and rehearsing a shopping list	(i) Street crossing cognitive performance (typing and crossing failures); (ii) street crossing motor performance (stay time, crossing speed); (iii) DT costs (linear mixed models effects comparing single and DT performances)	A virtual scenario, depicting a virtual street from the first-person perspective, was projected onto the screens. It consisted of city buildings grouped around a back alley and a tree-lined street with two lanes

DT, dual-task; VR, virtual reality.

**Table 2:** Summary of the included studies about CMDT training using VR systems.

**Study**	**Participants**	**Adopted technology**	**DT intervention**	**Session details**	**Virtual environment**	**Principal outcomes**	**Main results**
Zukowski et al. (2022)	Sixty Healthy older adults	GRAIL system: dual-belt treadmill positioned within and integrated with a 180-degree curved projection screen (semi-immersive)	Walking while identifying and gathering specified items and avoiding distractors	1 session for 30 min	Ocean boardwalk, Italian village street, plane runway	(i) Motor functions (walking speed, balance, functional mobility); (ii) DT (visuospatial reaction time while walking)	CMDT improved walking speed, response accuracy, and cognition throughout single and DT conditions. No significant group differences between the VR group and the no-VR group
Bruce et al. (2019)	Forty-two older Adults with and without Hearing Loss	iPad (non-immersive)	Aerobic training while holding discriminated fruits or vehicles on an iPad	6 weeks: 12 sessions, twice per week for 30 min	Figure at the center of a screen (e.g., fruits, vehicles)	(i) motor functions (postural control, mobility, balance); (ii) cognitive functions (auditory working memory)	The intervention improved motor functions, with no benefit to standing balance. Gains in cognitive functions were greater in the sequential VR-based CMDT group than in the simultaneous group, particularly among subjects without hearing loss. Participants with hearing loss were unaffected by the format
Hassandra et al. (2021)	Twenty-seven individuals with Mild Cognitive Impairment	Cycle-ergometer and HMD (immersive)	Cycling while solving numerical calculations	1 session of 20 min	Natural environment with a forest path, trees, grass, and mountains. The forest was populated with animals and flowers.	Feasibility, acceptability, usability, and tolerability of the training program	The main outcomes indicate that the intervention results feasible
Nayak et al. (2021)	Twenty-two Healthy older Adults	Treadmill in front of a display monitor, and an interactive computer game subsystem (non-immersive)	Walking or cycling while performing various cognitive activities, such as visual search and tracking of multiple targets, matching, and working memory tasks	12 weeks: 24 sessions, twice per week for 1 h	Stimuli from Big Fish Games (a rectangle object with a moving circle object and a soccer ball with a dotted sphere appeared on a black screen)	Feasibility (implementation, safety, acceptance, retention/compliance) of the training program	A high compliance rate and positive outcomes indicate that the training is feasible

The table categorizes the studies into two main groups: studies aimed at evaluating the efficacy of training interventions and those focused on aspects such as usability and feasibility. DT, dual-task; CMDT, cognitive-motor dual-task; VR, virtual reality.

### 3.1 CMDT assessment using VR systems

Seventeen studies examined VR-based CMDT assessment, predominantly targeting community-dwelling older adults with subclinical conditions rather than clinical conditions. Specifically, the majority focused on older adults with hearing impairment (Downey et al., 2023; Mohanathas et al., 2024; Tung Lau et al., 2016; Wu et al., 2014), subjective cognitive decline (Carr et al., 2019), elevated risk of falls (Gaspar et al., 2013; Zhou et al., 2019). Only two studies included clinical populations with memory impairment (Cook et al., 2013) and Parkinson's disease (Rosenfeldt et al., 2024). Three studies included community-dwelling older adults with health conditions Carr et al., (2020); Francis et al., (2015); Lewis et al., (2023). This distribution suggests VR-based CMDT assessment is currently positioned as a preventive screening tool rather than a diagnostic instrument. Indeed, the emphasis on sensory deficits and fall risk may indicate that CMDT performance is considered a sensitive early marker of functional decline in otherwise healthy aging.

Authors demonstrated a strong preference for immersive VR configurations (12/17 studies). Eight studies employed fully immersive systems such as the CAVE Automatic Virtual Environment system (CAVE) was utilized by Ehlers et al. (2017) and Nagamatsu et al. (2011), Street LAB system (Carr et al., 2019, 2020; Downey et al., 2023; Mohanathas et al., 2024; Tung Lau et al., 2016), and Head-Mounted Display (HMD) (Lewis et al., 2023; Rosenfeldt et al., 2024; Souza Silva et al., 2020). Semi-immersive systems occurred in six studies utilizing either simulator driving platforms (Cook et al., 2013; Gaspar et al., 2013; Wu et al., 2014) or instrumented treadmills with projection screens (Francis et al., 2015; Mack et al., 2023; Stojan et al., 2023). Moreover, assessment protocols overwhelmingly favored realistic environmental situations. The most common paradigm simulating urban navigation with traffic (Carr et al., 2019, 2020; Downey et al., 2023; Ehlers et al., 2017; Mack et al., 2023; Mohanathas et al., 2024; Nagamatsu et al., 2011; Stojan et al., 2023; Tung Lau et al., 2016). These environments provided virtual street-crossing scenarios where participants had to safely cross streets while conversing with hands-free phones or a 6-lane street intersection in Toronto, where participants engaged in street crossing tasks while performing concurrent cognitive activities such as word recognition or dichotic listening tasks. Five studies simulates daily activities such as grocery shopping Lewis et al., (2023); Rosenfeldt et al., (2024), subway navigation Souza Silva et al., (2020), and driving scenarios Cook et al., (2013); Gaspar et al., (2013); Wu et al., (2014). One study employed stylized, game-like stimuli using a forest with wasps, prioritizing experimental control over realism. These data suggest that the field has converged on functional simulation as the goal standard for assessment, with the street crossing as a canonical task.

Task configurations showed various motor-cognitive combinations. Tasks used in nine of the 17 studies could be classified as “walking while” tasks Ehlers et al., (2017); Francis et al., (2015); Lewis et al., (2023); Mack et al., (2023); Nagamatsu et al., (2011); Rosenfeldt et al., (2024); Souza Silva et al., (2020); Stojan et al., (2023); Tung Lau et al., (2016). These paradigms combined a simple walking task as the motor component, usually performed on an instrumented treadmill, with a concurrent cognitive task, including conversing with a hands-free phone Ehlers et al., (2017); Nagamatsu et al., (2011), words recognition Tung Lau et al., (2016), counting backwards Francis et al., (2015), serial threes and color word Stroop task Stojan et al., (2023), reading and memorizing text messages Souza Silva et al., (2020), and virtual shopping tasks Lewis et al., (2023); Mack et al., (2023); Rosenfeldt et al., (2024). The remaining eight studies used alternative motor-cognitive combinations. Five studies adopted standing, postural, or balance tasks as the motor component paired with various cognitive challenges, such as listening tasks Carr et al., (2019), (2020); Mohanathas et al., (2024), serial subtraction tasks de Rond et al., (2021), auditory tasks Downey et al., (2023). Three studies employed driving simulation as the motor component: Cook et al. (2013) associated it with story listening, rehearsing, and recalling; Wu et al. (2014) with speech recognition, while Gaspar et al. (2013) with a 1-back task. “Walking while” paradigm appears as a standardized framework that facilitates comparison across studies and aligns with traditional assessments like the TUG and WWTT.

The most significant methodological weakness emerged in DT cost calculations. The statistical comparison approach was the most used (10/17 studies). It was used inferential statistics (ANOVA, linear mixed models) to compare single-task vs. DT conditions, without computing DT cost ratio Carr et al., (2019), (2020); Cook et al., (2013); Francis et al., (2015); Gaspar et al., (2013); Mack et al., (2023); Mohanathas et al., (2024); Nagamatsu et al., (2011); Tung Lau et al., (2016); Wu et al., (2014). On the other hand, DT cost ratio was calculated in 7 of the 14 studies. They computed proportional or absolute change metrics; however, they used non-standardized formulas to quantify the magnitude of cognitive-motor interference Carr et al., (2020); Downey et al., (2023); Ehlers et al., (2017); Lewis et al., (2023); Rosenfeldt et al., (2024); Souza Silva et al., (2020); Tung Lau et al., (2016). Computational formulas varied, with some using percentage change calculations and others using absolute difference scores, though several studies did not explicitly specify their method. These approaches are mathematically and conceptually non-interchangeable Leone et al., (2015), limiting cross-study comparison and consensus of standardized metrics.

### 3.2 CMDT training using VR systems

Four studies examined VR-based CMDT training. This limited body of research contrasts with the 17 assessment studies revealing a substantial imbalance in the field.

The research spanned populations, including healthy older adults Nayak et al., (2021); Zukowski et al., (2022), and adults with and without hearing loss Bruce et al., (2019). Unlike assessment studies that focused predominantly on subclinical populations, training research included both preventive applications and therapeutic interventions. This might reflect the field's exploratory phase.

Authors demonstrated a technological preference for non-immersive VR systems (2/4 studies). Particularly, they employed a treadmill with a frontal flat screen displaying computer games (Nayak et al., 2021). Activities were controlled with an inertial mouse tied to the participant's head during the interaction with stylized stimuli (rectangular objects, circular targets from commercial Big Fish Games). Bruce et al. (2019) used an iPad to display interactive cognitive exercises (i.e., discrimination task) while patients performed various physical exercises (i.e., aerobic training) (Bruce et al., 2019). Semi-immersive systems occurred in one study using a treadmill with a large projection screen displaying the virtual environment directly in front of the participants (Zukowski et al., 2022). Participants gathered pizza ingredients while avoiding obstacles in a virtual Italian village street with a wooden cart avatar controlled by body movements. Hassandra et al. (2021) provided a fully immersive HMD configuration with the VRADA: participants cycled while HMD simulated an outdoor natural virtual environment (with a forest path, trees, grass, and mountains) where participants had to solve math quizzes. Training protocol favored abstract stimuli and simplified environments, with two of the four studies employing stylized stimuli from commercial game objects such as geometric shapes (e.g., rectangles and circles), fruits, and vehicles on plain backgrounds (Bruce et al., 2019; Nayak et al., 2021). On the other hand, two studies replicate an Italian village street with simplified graphics (Zukowski et al., 2022) and a forest path with mathematical overlays (Hassandra et al., 2021). No training study replicated the high fidelity of real environments used in assessment protocols.

Moreover, training protocols presented substantial variability in terms of duration and frequency. Two studies conducted a single-session training of 20 min (Hassandra et al., 2021; Zukowski et al., 2022) as a feasibility test and efficacy evaluation, respectively. In contrast, extended programs lasting 6 (Bruce et al., 2019) or 12 weeks (Nayak et al., 2021) with 2 weekly sessions of 30–60 min, respectively. The field lacks consensus on the minimal effective dosage. Single-session studies demonstrated acute effects on gait and cognition (Zukowski et al., 2022), while extended programs showed improvements in motor functions and working memory (Bruce et al., 2019; Nayak et al., 2021). However, without standardized protocols or dose-response analyses, optimal training parameters remain undefined. No studies included follow-up evaluations.

Task configurations showed various CMDT. Tasks used in two of the four studies could be classified as “walking-based” paradigms. These paradigms combined a walking task as the motor component, performed on a treadmill, with a concurrent cognitive task, including visual search (Nayak et al., 2021; Zukowski et al., 2022). One study employed a “cycling-based” paradigm with a stationary bike and mathematical calculation tasks. Bruce et al. (2019) combined aerobic training with an iPad-based discrimination task.

We noted that studies evaluated two primary domains: intervention efficacy (measuring motor functions, cognitive abilities, and DT performance), and user experience, including feasibility, acceptability, and tolerability (Hassandra et al., 2021), and adherence rates (Nayak et al., 2021). Results revealed positive findings across interventions that improved walking speed, dual task accuracy (Zukowski et al., 2022), motor functions, and working memory (Bruce et al., 2019) with high acceptability ratings and high compliance among older adults (Hassandra et al., 2021; Nayak et al., 2021).

## 4 Discussion

This scoping review mapped the current VR-based CMDT applications for assessment and training in older adults, with specific attention to how immersion level, ecological validity, and practical implementation constraints shape current approaches. Seventeen studies employing VR for CMDT assessment and four studies applying VR for CMDT training, evidencing a markedly more mature body of work in assessment. Assessment research leveraged immersive VR to recreate realistic daily mobility challenges and manipulate cognitive-motor load in a controlled yet ecologically valid environment. In contrast, the evidence for training seems to remain preliminary.

A central finding of this scoping review is the disconnect between the maturity of the assessment field and the early developmental stage of training research. Assessment studies predominantly employed immersive, high-fidelity VR scenarios, including street crossings, supermarkets, and public transportation settings simulated in CAVE environments, StreetLab platforms, and HMDs. Conversely, training studies relied mostly on non-immersive or semi-immersive setups, such as treadmills positioned in front of flat screens displaying virtual environments, with only one study implementing a fully immersive HMD configuration (Hassandra et al., 2021). This technological divergence likely reflects different priorities. Assessment studies emphasize naturalistic simulation and detailed measurement, whereas training research must balance ecological fidelity with feasibility, accessibility, and tolerability across repeated sessions despite potentially limiting ecological validity and, therefore, real-world transfer. Indeed, while immersive VR environments represent the vertex of ecological and engaging perspectives (Bauer and Andringa, 2020; Ventura et al., 2019), their implementation may induce cybersickness, fatigue, or cognitive overload, especially in older adults, limiting their suitability for extended interventions. Moreover, aging people may benefit more from less immersive solutions that offer a manageable and appropriate level of sensory engagement while maintaining therapeutic efficacy (Realdon et al., 2019). Non-immersive systems also enhance the scalability of interventions and facilitate home-based adoption, improving accessibility and feasibility. Moreover, there are additional issues in delivering structured stimuli, which largely stem from technical challenges in VR implementation, particularly complex technical setups and the development of sophisticated 3D interfaces (Riva et al., 2020), which can be both time-consuming and resource-intensive. Approaches such as 360° video may offer a scalable middle ground (Mancuso et al., 2024), but no included study adopted this strategy. This is particularly noteworthy given that 360° media may offer potential advantages in terms of development cost, technical accessibility, and ecological realism compared to computer-generated VR environments. The absence of this technology in CMDT research may reflect the field's current developmental stage or technical barriers to integrating dynamic cognitive-motor tasks within 360° environments. However, this represents a clear opportunity for future research, as 360° media could provide a practical middle ground between ecological validity and implementation feasibility, particularly valuable for resource-constrained clinical settings or home-based interventions where fully immersive systems may be impractical. Although these divergences, the training studies reported improvements in gait, cognitive performance, or DT cost, and generally positive user acceptance. These findings indicate feasibility and potential benefits, but they do not yet constitute robust evidence of efficacy. However, none included follow-up assessment, limiting conclusions regarding long-term retention or causal efficacy.

A further gap concerns the populations. Our findings reveal that the majority of studies focusing on healthy older adults or those with early cognitive decline (Mild Cognitive Impairment), sensory deficits (hearing loss), and elevated fall risk highlight a substantial gap in the literature regarding VR-based CMDT applications for large clinical populations. This pattern suggests that current research emphasizes preventive rather than interventional approaches, where paradigms such as walking-while-talking or walking-while-calculating were used in the early detection of cognitive-motor decline, potentially facilitating timely interventions before significant functional impairment manifests (Mancioppi et al., 2021).

Assessment studies displayed inconsistencies primarily in the calculation of DT outcomes, reflecting the lack of consensus on standardizing assessment methods (Leone et al., 2015). These divergent methods complicate cross-study comparisons and the synthesis of findings. On the other hand, training studies showed broad heterogeneity across populations, VR configurations, task structures, dosage, and outcome metrics. No two training studies were methodologically comparable, and most lacked control groups, limiting confidence in observed improvements. This variability suggests that the field is still in a developmental phase regarding optimal training parameters. Nevertheless, our findings suggest that twice-weekly sessions may represent a reasonable approach, aligning with literature indicating that 2–3 sessions per week allow adequate recovery between sessions while minimizing fatigue risk (Erickson et al., 2019). Despite these substantial differences, our studies collectively suggest that VR-based CMDT training represents a feasible and potentially effective approach for enhancing both physical and cognitive functions in older adults, with promising short-term outcomes that warrant further investigation through more robust and longer-duration studies. Importantly, none of the training studies included follow-up assessments, representing a significant limitation for understanding the long-term retention of treatment effects. These findings highlight the absence of a unified translational pipeline from VR-based CMDT assessment to intervention design.

Although user experience is critical for the feasibility and scalability of VR-based interventions, it was systematically underreported. Only a minority of studies used validated instruments, and most provided minimal qualitative comments. This gap limits conclusions about tolerability, accessibility, and clinical implementation. However, literature suggests that user experience is a critical preliminary phase for developing efficacious and accessible VR protocols (Broekhuis et al., 2021; Tuena et al., 2023), because intervention parameters should be carefully calibrated on objectives and target populations' characteristics. However, our results reveal a limited body of research focusing on the user experience related to VR solutions, which could raise concerns about whether these technologies achieve their intended benefits.

Important final considerations concern the operationalization of CMDT itself. Although CMDT is traditionally defined as the concurrent performance of one cognitive and one motor task with distinct and separable goals (McIsaac et al., 2015), maintaining this clear dissociability—which theoretically differentiates actual DT from other complex activities—becomes increasingly challenging in naturalistic settings where cognitive and motor components are inherently integrated. For this reason, studies incorporating real-life simulations (e.g., bathroom activities or driving) were excluded from systematic analysis, as the separation between cognitive and motor components was unclear (Cassavaugh and Kramer, 2009; Park et al., 2020). Similarly, studies combining motor activities, such as cycle ergometer use, with cognitive tasks involving attention, spatial orientation, or obstacle avoidance were excluded, as motor actions directly served cognitive goals rather than maintaining distinct aims (Kwan et al., 2024; Mirelman et al., 2013; Pelosin et al., 2022). Such tasks more accurately represent integrated or complex motor tasks rather than a proper DT. While some included scenarios (e.g., driving) involving ecological complexity, they maintained the core CMDT structure with independent and separate goals. This differs fundamentally from integrated activities, where cognitive and motor processes served a unified objective. However, this ambiguity points to the need for a more refined conceptual framework acknowledging the interdependence of cognitive and motor processes in daily tasks while preserving the DT paradigm's utility.

## 5 Conclusions

Our findings underscore the need for research practices that more closely align assessment insights with intervention development. Future studies would benefit from greater standardization of DT cost calculations and clearer reporting of key implementation details, including hardware specifications, immersion level, task structure, and safety procedures. Developing hybrid or 360° media solutions may help bridge between high ecological validity and practical feasibility, enabling more tolerable, scalable, and context-rich protocols for older adults.

From a clinical perspective, semi-immersive systems may currently offer the most favorable balance between engagement and tolerability for repeated CMDT training, while fully immersive environments may be better suited for comprehensive assessments that require high ecological fidelity. Existing immersive assessment protocols already provide validated templates that clinicians can adapt for functional evaluation. Although the evidence base remains limited, the available studies suggest that training administered two to three times per week may be feasible and beneficial in the short term, although long-term effects remain unknown. Finally, technology developers play a key role in the future evolution of VR-based CMDT tools. Prioritizing user-centered design, reducing cybersickness triggers, and optimizing interfaces for ease of calibration and setup will be essential for enabling clinical and home-based deployment. Overall, advancing VR-based CMDT research will require coordinated methodological refinement, more robust studies, and closer collaboration across scientific, clinical, and technological domains to ensure that VR-based solutions can progress from experimental prototypes to clinically impactful tools.

This scoping review had several limitations. We included heterogeneous studies with different methodologies, VR configurations, intervention parameters, and outcome measures. While this offered a broad perspective on the topic, it also induced potential biases and challenges in synthesizing the results. Additionally, we restricted our search to three databases and to English-language publications, which may have led to the omission of relevant studies published elsewhere, potentially excluding emerging or unpublished VR-based CMDT applications. Our age criterion (average age ≥65 years) was based on conventional gerontological definitions of older adulthood. Still, it may have excluded relevant studies with mixed-age samples or “young-old” populations (60–64 years) where significant portions of participants were below 65 years. This approach, while providing a clear operational threshold, may potentially limit the comprehensiveness of our mapping, particularly for clinical populations where CMDT impairments typically manifest in the sixth decade of life. Future reviews might consider more flexible age criteria or explicit inclusion of age distribution data to capture the full spectrum of aging-related CMDT research. Furthermore, in line with scoping review methodology, we did not conduct a formal assessment of study quality or risk of bias. While this approach is consistent with established scoping review guidelines (Munn et al., 2018; Tricco et al., 2018), which prioritize breadth of mapping over evaluation of evidence strength, it limits our ability to draw strong conclusions about intervention effectiveness or to provide clinical recommendations. The evidence base we mapped consists largely of feasibility studies, pilot investigations, and cross-sectional assessments, with limited high-quality randomized controlled trials. As a result, our synthesis provides a comprehensive overview of available VR-based CMDT approaches and identifies key methodological characteristics and research gaps, but cannot establish definitive conclusions about the efficacy or clinical utility of these interventions. Future systematic reviews incorporating formal quality appraisal and meta-analysis would be valuable to establish the effectiveness of VR-based CMDT interventions and inform evidence-based clinical guidelines. Future research may also benefit from more standardized approaches focusing on subsets of methodologically similar studies to address these limitations.
